# Statins and the risk of gastric cancer in diabetes patients

**DOI:** 10.1186/1471-2407-12-596

**Published:** 2012-12-13

**Authors:** Jeeyun Lee, Soo Hyeon Lee, Kyu Yeon Hur, Sook Young Woo, Sun Woo Kim, Won Ki Kang

**Affiliations:** 1 Division of Hematology-Oncology, Department of Medicine, Samsung Medical Center, Sungkyunkwan University School of Medicine, 50 Irwon-dong Gangnam-gu, Seoul, 135-710, Korea; 2 Division of Endocrinology, Department of Medicine, Samsung Medical Center, Sungkyunkwan University School of Medicine, 50 Irwon-dong Gangnam-gu, Seoul, 135-710, Korea, Korea; 3 Yonsei University College of Medicine, 250 401 Seongsanno, Seodaemun-gu, Seoul, 120-752, Korea; 4 Biostatistics, Samsung Medical Center, Sungkyunkwan University School of Medicine, 50 Irwon-dong Gangnam-gu, Seoul, 135-710, Korea

**Keywords:** Gastric cancer, Statins, Risk

## Abstract

**Background:**

Several studies have suggested a cancer risk reduction in statin users although the evidence remains weak for stomach cancer. The purpose of this study was to use an exact-matching case–control design to examine the risk of gastric cancer associated with the use of statins in a cohort of patients with diabetes.

**Methods:**

Cases were defined as patients with incident gastric cancer identified by International Classification of Diseases 16.0 ~ 16.9 recorded at Samsung Medical Center database during the period of 1999 to 2008, at least 6 months after the entry date of diabetes code. Each gastric cancer case patient was matched with one control patient from the diabetes patient registry in a 1:1 fashion, blinded to patient outcomes.

**Results:**

A total of 983 cases with gastric cancer and 983 controls without gastric cancer, matched by age and sex, were included in the analysis. The presence of prescription for any statin was inversely associated with gastric cancer risk in the unadjusted conditional logistic regression model (OR: 0.18; 95% CI: 0.14 – 0.24; P < .0001). Multivariate analysis using conditional logistic regression with Bonferroni’s correction against aspirin indicated a significant reduction in the risk of gastric cancer in diabetes patients with statin prescriptions (OR: 0.21; 95% CI: 0.16 – 0.28; P < .0001). After adjustment for aspirin use, a longer duration of statin use was associated with reduced risk of gastric cancer, with statistical significance (P<.0001).

**Conclusions:**

A strong inverse association was found between the risk of gastric adenocarcinoma and statin use in diabetic patients.

## Background

Gastric cancer is the leading cause of cancer death worldwide, with an incidence of 18.9/100,000 per year and a mortality rate of 14.7/100,000 per year [1]. It is also the most common cancer type in Korea. Despite the worldwide prevalence of gastric cancer, its chemoprevention has been relatively poorly studied.

Several large-scale epidemiology studies have highlighted the potential anti-tumor effect of statins on various solid tumors. Statins are synthetic 3-hydroxy-3-methylglutaryl coenzyme A (HMG-CoA) reductase inhibitors that are commonly used drugs for the treatment of hypercholesterolemia. Statins inhibit the rate-limiting step of the mevalonate pathway that produces mevalonic acid, the precursor for the biosynthesis of isoprenoid molecules such as cholesterol, dolichol, and ubiquinone. Mevalonate-derived prenyl groups, farnesyl pyrophosphate (FPP), and geranylgeranyl pyrophosphate (GGPP) facilitate essential intracellular functions of various proteins such as Ras and Rho [1-3]. One of the first large-scaled epidemiologic studies, the MECC (The Molecular Epidemiology of Colorectal Cancer) study, demonstrated that the use of statins for more than 5 years was associated with a 47 percent relative reduction in the risk of colorectal cancer, after adjustment for other known risk factors such as age, sex, ethnic group, hypercholesterolemia, history of colorectal cancer in a first-degree relative, and level of vegetable consumption [4]. Although the results from these studies support the hypothesis that statins may reduce the risk of cancer, overall results from observational studies still remain inconclusive, since subsequent studies showed no significant association between statins and cancer occurrence [5].

Recently, several interesting studies have analyzed the risk of cancer in patients with diabetes mellitus (DM) who were on statin therapy. Among these studies, the Hong Kong group analyzed a DM cohort of 5,276 patients and found a decreased risk of cancer in non-statin users and co-presence of high LDL cholesterol [6]. In contrast, statin non-users with low LDL cholesterol and albuminuria had a 4.9-fold risk for any cancer when compared with statin users [6]. The same research group also demonstrated that statins reduced cancer risk in DM patients, potentially via normalizing hydroxymethylgutaryl-CoA reductase (HMGCR) and the insulin-like growth factor-1 (IGF-1) signaling pathway [7]. In addition, statin use was associated with a significant 38% reduction in the risk of hepatocellular carcinoma among DM patients [8]. For gastric cancer, a recent meta-analysis demonstrated a potential protective effect of statins, with median risk ratio of 0.59 (range, 0.40 – 0.88) [9]. A nested case control study reported an odds ratio of 0.86 (95% CI, 0.72 – 1.02) for stomach cancer based con 1,992 cases and 8,279 controls [10]. Nevertheless, the evidence remains weak and no large-scaled epidemiologic studies have yet been conducted to specifically analyze the risk of gastric cancer and its association with statin use.

We undertook this study to examine the risk of gastric cancer associated with the use of statins using an exact matching case–control design nested in a cohort of patients with diabetes. We also investigated the potential association between duration of statin use and the risk of gastric cancer.

## Methods

We first identified a large cohort of patients with DM who were treated at Samsung Medical Center. In this study, study cases were defined as patients who were diagnosed with gastric cancer at least 6 months after the entry date of the diabetes code. Gastric cancer was identified by International Classification of Diseases 16.0 ~ 16.9 recorded at Samsung Medical Center database during the period of 1999 to 2008. Cases were excluded if gastric cancer was diagnosed shortly after or before the diagnosis of diabetes: any gastric cancer cases diagnosed within 6 months of the diagnosis of diabetes were excluded from this study. All included cases were pathologically or cytologically diagnosed at our center. Each gastric cancer case patient was matched with one control patient from the diabetes patient registry, in a 1:1 fashion that was blinded to patient outcomes. Matching variables were age (exact) and sex at an index date, which was the date of the gastric cancer diagnosis for each case. We excluded patients who had no prescription record available in our database or those who were followed up at our hospital for less than 1 year.

### Exposure to statins

Statin prescriptions were collected from our electronic prescription record system. Statins included simvastatin, atorvastatin, lovastatin, rosuvastatin, pitavastatin, pravastatin, and cerivastatin. We collected the dates of filled prescriptions, daily dose, number of days supplied, and the number of pills per prescription. In the cases of statin prescribed before visiting Samsung Medical Center, we utilized the electronic drug identification system at our center, which identifies the drug and dosage to hospital pharmacists upon consultation. As a routine practice, we consult all of the outside prescription drugs with hospital pharmacists to verify identification of drugs. We collected prescription information on aspirin, nonstatin lipid-lowering medications (cholestyramine, colestipol, and niacin), and triglyceride-lowering medications (clofibrate, fenofibrate, and gemfibrozil). Statin users were defined as those who had statin filled prescriptions for at least 6 months.

### Clinical data validation

All of the inpatient and outpatient charts at Samsung Medical Center, including the drug prescription system, are currently electronic. After identification of cases and controls, the following clinical data were collected from the electronic medical chart system: date of diagnosis of gastric cancer, stage according to AJCC 2002, Lauren classification (intestinal, diffuse, and mixed), pathology, smoking status (never smoker, smoker, not checked), location, and *Helicobacter pylori* status.

### Statistical analyses

Descriptive data are presented as means and proportions, as well as by frequency, for continuous data and categorical data, respectively. For univariate analysis, conditional logistic regression analysis was used to identify the risk effect for gastric cancer because of matched data. Conditional multiple logistic regression analysis was used to detect the effect of statin use for gastric cancer after adjusting for aspirin use.

We investigated the statin duration-effect for gastric cancer using conditional logistic regression by evaluating the effect of the duration of any statin prescriptions examined in the following categories: 0.5 – 1.0 year, 1.0 – 1.5 years, 1.5 – 2.0 years, greater than 2.0 years. The filled prescription record for any statin during the designated time period included both in-hospital prescription and outside hospital filled prescriptions identified in the medical record system. Bonferroni’s correction was used to correct inflation type I error due to multiple testing. For all analyses, the a priori level of significance was 0.05. All data analyses were conducted using SAS software, version 9.1.3 (SAS Institute, Inc., Cary, NC).

This study was reviewed and approved by the Samsung Medical Center Institutional Review Board in accordance with the Declaration of Helsinki.

## Results

The diabetes cohort of 12,001 patients treated at Samsung Medical Center between 1999 and 2008 included 1,106 potentially eligible patients in whom gastric adenocarcinoma (including signet ring cell carcinoma) was identified during the study period. Of those 1,106 patients, 123 patients were excluded from the analysis due to lack of prescription records or insufficient clinical data for gastric cancer. Thus, 983 cases with gastric cancer and 983 controls without gastric cancer, matched by age and sex, were included in the final analysis. All of the patients were of Korean ethnicity. The mean age was 62.6 years in each cohort and 76.5% of the study population were male (Table 1). Owing to the exact matched pair analysis, the distributions of age and sex were identical between the two cohorts. The prescription data for statin and aspirin use are provided in Table 1. The mean duration between the date of entry into the diabetes cohort and the date of gastric cancer index was 673 days. 

**Table 1:** Demographic factors and filled prescription data in cases with advanced gastric cancer (AGC) and matched controls

**Variables**	**AGC (n=983)**		**Control (n=983)**	
	**N**	**%**	**N**	**%**
Mean age, y	62.6		62.6	
Sex				
Men	752	76.5	752	76.5
Women	231	23.5	231	23.5
**Statin user**	**99**	**10.1**	**367**	**37.3**
Atorvastatin	31	31.3	143	39.0
Simvastatin	27	27.3	67	18.2
Rosuvastatin	11	11.1	59	16.1
Pravastatin	12	12.1	48	13.1
Pitavastatin	4	4.0	18	4.9
Others	14	14.1	32	8.7
Statin exposure duration (year)				
0 - < 0.5	884	89.9	616	62.7
0.5 – 1.0	38	3.9	71	7.2
1.0 – 1.5	12	1.2	61	6.2
1.5 – 2.0	13	1.3	41	4.2
>2.0	36	3.7	194	19.7
Aspirin				
Yes	184	18.7	347	35.3

### Statin Use and the risk of gastric cancer

The presence of prescription for any statin was inversely associated with gastric cancer risk in the unadjusted conditional logistic regression model (OR: 0.18; 95% CI: 0.14 – 0.24; P < .0001; Table 2). After adjustment for a potential confounder (aspirin), the association between statins and the reduced risk of gastric cancer remained significant (adjusted OR for aspirin: 0.61; 95% CI: 0.48 – 0.77; Table 2). Multivariate analysis using conditional logistic regression with Bonferroni’s correction against aspirin indicated a significant reduction in the risk of gastric cancer in diabetes patients with statin prescriptions (OR: 0.21; 95% CI: 0.16 – 0.28; P < .0001; Table 2). 

**Table 2:** Effect of statin use on the incidence of gastric cancer in diabetic patients

**Univariable analysis**				
**Variables**	**Case**	**Control**	**P-value**	**OR(95% CI)**
Statin use	99	367	< .0001	0.183 (0.139 – 0.241)
Aspirin	184	347	< .0001	0.418 (0.337 – 0.518)
Other lipid lowering agents	154	221	< .0001	0.148 (0.073 – 0.297)
**Multivariable analysis**				
**Variable**	**Case**	**Control**	**P-value**	**OR (95% CI)**
Statin use	99	367	< .0001	0.211 (0.159 – 0.281)
Aspirin	184	347	< .0001	0.608 (0.478 – 0.773)
Other lipid lowering agents	154	221	< .0001	0.156 (0.073 – 0.337)

Conditional logistic regression analysis was used.

Conditional multiple logistic regression analysis was used.

*Abbreviations: *OR* odds ratio.

### Duration of statin Use and the risk of gastric cancer

We next examined the impact of duration of statin use on gastric cancer risk. We subgrouped the patient cohort according to duration of statin use. Multivariate analysis using conditional logistic regression, adjusting for aspirin, indicated a significant reduction in the risk of gastric cancer in diabetes patients in statin users group when compared with the nonexposed group (Table 3). Intriguingly, patients who had a longer duration of statin prescriptions had more reduced risk for gastric cancer. Diabetes patients with less than 1 year of statin use before the index date had an odds ratio (OR) of 0.45; however, patients with statin use of more than 2 years had an OR of 0.154 (95% CI: 0.09 – 0.26; P < .0001). We analyzed the duration of statin use and the risk of gastric cancer by performing a trend test using conditional logistic regression analysis. After adjustment for aspirin, we found that a longer duration of statin use reduced the risk of gastric cancer and the difference was statistically significant (P<.0001). 

**Table 3:** Duration of statin use and the risk of gastric cancer in diabetic patients

**univariable analysis**				
**Variable (Statin duration)**	**Case**	**Control**	**P-value**^**+**^	**OR (95% CI)**^**+**^
0.5-1.0	38	71	< .0001	0.375 (0.216 – 0.651)
1.0-1.5	12	61	< .0001	0.138 (0.059 – 0.319)
1.5-2.0	13	41	< .0001	0.152 (0.058 – 0.400)
≥2.0	36	194	< .0001	0.132 (0.080 – 0.219)
**multivariable analysis**				
**Variable (Statin duration)**	**Case**	**Control**	**P-value**	**OR (95% CI)**
0.5-1.0	38	71	0.0016^+^	0.446 (0.252 – 0.790) +
1.0-1.5	12	61	< .0001^+^	0.143 (0.061 – 0.338) +
1.5-2.0	13	41	< .0001^+^	0.178 (0.067 – 0.474) +
≥2.0	36	194	< .0001^+^	0.154 (0.091 – 0.260) +
Aspirin	184	347	< .0001	0.618 (0.454 – 0.843)
Other lipid lowering agents	154	221	< .0001	0.144 (0.053 – 0.392)

(reference category: Unexposed group).

Conditional logistic regression analysis was used.

^+^ ; Bonferroni’s correction was used to adjust inflation type I error due to multiple testing.

(reference category: Unexposed group).

Conditional multiple logistic regression analysis was used.

^+^ ; Multiple conditional logistic regression with Bonferroni’s correction.

*Abbreviations: *OR* odds ratio.

### Statin Use and clinical features of gastric cancer

**Figure 1:**
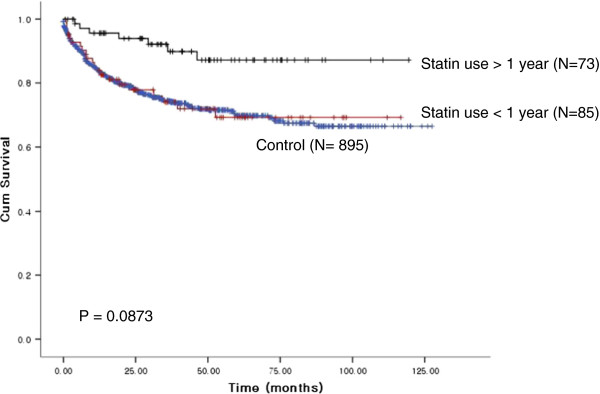
Survival according to statin use.

Among the 983 cases with gastric cancer, 99 patients had begun statin therapy > 6 months prior to the diagnosis of gastric cancer. We performed a further association analysis to characterize the clinical features of statin users among gastric cancer patients (Table 4). Statin use was not significantly associated with smoking status, Lauren classification, location, or the presence of *Helicobacter pylori*. Intriguingly, the proportion of localized disease was significantly higher (81.8% vs. 74.0%; statin user vs. non-user; P = 0.0400) in statin users (81.8%) than in non-users (74.0%) (P=0.0400) (Table 4). In addition, a trend toward favorable survival outcome was evident in stain users when compared with non-statin user gastric cancer patients (5-year OS, 89.3% vs. 73.0%; statin user vs*.* non-user; P=0.0873, Figure 1). 

**Table 4:** Statin use in gastric cancer patients

**Variables**	**Statin User**		**Non-Statin User**		***P *value**
	**N**	**%**	**N**	**%**	
**Sex**					
Men	71	71.7	681	77.0	.2365
Women	28	28.3	203	23.0	
**Lauren classification**					
Intestinal	22	22.2	283	33.8	.1351
Diffuse	67	67.7	518	61.9	
Mixed	4	4.0	36	4.3	
Missing information	6	6.0	47	5.3	
**Pathology**					
W/D adenocarcinoma	26	26.3	142	16.0	.1461 (adenoca *vs* signet ring)
M/D adenocarcinoma	39	39.4	357	40.3	
P/D adenocarcinoma	21	21.2	251	28.3	
Signet ring cell carcinoma	13	13.1	134	15.4	
**Smoking**					
Never-smoker	51	51.5	391	44.2	.3037
Smoker	47	47.5	472	53.4	
Missing information	1	1	21	2.4	
**Location**					
Cardia	6	6.1	51	5.8	
Body ~ antrum	91	91.9	817	92.5	.9660
Whole, multifocal	2	2.0	15	1.7	
Missing information	0	0	1	0.1	
**Helicobacter Pylori**					
Present	44	44.4	331	37.4	.1897
Absent	41	41.4	405	45.8	
Missing information	14	14.2	148	16.7	
**Stage**					
Localized (stage I/II)	81	81.8	642	72.6	.0400
Advanced (stage III/IV)	16	16.2	226	25.6	
Missing information	2	2.0	16	1.8	

*Abbreviations: *W/D* well differentiated, *M/D* moderately differentiated, *P/D* poorly differentiated.

## Discussion

A potential benefit of statin use has been suggested for various tumor types, including colorectal cancer [4,11], multiple myeloma [12], prostate cancer, breast cancer [13,14], hepatocellular carcinoma [8], and lymphoma [15]. This matched case–control study represents the first study to indicate the existence of a strong inverse association between the risk of gastric adenocarcinoma and statin use in diabetic patients. A trend toward stronger risk reduction was seen with longer duration of statin prescriptions. The risk reduction observed with statin use was about 78%, based on multivariate analysis. No significant correlations were noted between gastric cancer risk and prescriptions for aspirin.

Emerging evidence supports the potential role of statins as anti-cancer drugs in several tumor types. Statins are synthetic 3-hydroxy-3-methylglutaryl coenzyme A (HMG-CoA) reductase inhibitors and are commonly used drugs for the treatment of hypercholesterolemia. In our previous report, we demonstrated that a low dose lovastatin (equivalent to cardiovascular dose) induced G1 phase cell cycle arrest and cell senescence via RhoA modulation in cancer cells [16]. In addition, we reported results from a phase II study that combined a cardiovascular dose of simvastatin and standard FOLFIRI (irinotecan, infusional 5-fluorouracil, leucovorin) as treatment for metastatic colorectal cancer [17]. Lastly, we recently demonstrated that the addition of 0.2 μM simvastatin (equivalent to the human cardiovascular dose) to cetuximab significantly enhanced antitumor activity in KRAS mutant colon cancer cells, but not in BRAF^V600E^ mutant colon cancer cells [18].

The insulin and IGF1 signals triggered through the insulin receptors (IRs) and IGF1 receptor (IGF1R), respectively, result in activation of the phosphotidylinositol 3-kinase/Akt signaling pathway and protein kinase C. The IGF-1 and insulin signaling pathways are now known to play important roles in tumor cell growth that is correlated with diabetes risk and cancer [19]. The potential anti-tumor effect of statins has been reported for multiple IGF-1-dependent malignancies [20-22]. The strong inverse correlation between statin use and colorectal cancer [11], hepatocellular carcinoma [8], and other types in patients with diabetes [6,7] led us to survey the risk of gastric cancer in this context. Previous research has shown obesity, high body mass index, and low plasma adiponectin levels to have an association with an increased risk of gastric cancer, although the evidence is still inconclusive [23-25]. However, statins may plausibly exert an anti-tumor effect by modulating the IGF pathway in gastric cancer, especially in the subgroup of patients with diabetes. The risk of gastric cancer in statin users among diabetes patients should be prospectively confirmed for its definite role as a chemopreventive agent.

The strengths of our study would be its large patient cohort, the diagnosis of gastric cancer at a single center, statin exposure data collected from a single database, and the exact matched case–control paired analysis. Due to the importance of pathologic classification of gastric cancer, we excluded all cases which were not gastric adenocarcinoma or signet ring cell carcinoma. The statin exposure data were retrieved from our electronic medical record system, which comprises all inpatient and outpatient clinics. Hence, the prescription database is relatively accurate. Nevertheless, our study is limited by the observational retrospective nature of its study design. Other tentative risk factors for gastric adenocarcinoma, such as BMI, adenopectin levels, or dietary factors, were not available for correlative analyses. The adjusted odds ratio for gastric cancer was relatively low, conferring about a 70 ~ 80% risk reduction by statin use in diabetic patients in our study. This odds ratio is lower than those reported for other cancer types, which ranged from 20 – 60% risk reduction [4,8,11]. The retrospective nature of the analysis also could present potential confounders such as prescription bias for statins in the gastric cancer patient cohort. However, the matched case–control pairs were identified from a large patient pool with diabetes, which may minimize this type of bias, especially by eliminating those cases with diabetes entry after the index date for gastric cancer. Another potential bias for our observations would be the inherent bias from our patient population, all of whom visited a larger tertiary hospital in Korea instead of a private clinic for diabetes control. This patient pool may be more attentive to their health condition than others who visited the tertiary hospital for gastric cancer treatment, and thus might have led to an increased detection rate for statin use in diabetes group. Hence, our analysis should be cross-validated in other hospital settings. In addition, our observation that lower incidence of gastric cancer in statin users might be confounded by LDL cholesterol. Several epidemiological studies have reported that low plasma LDL cholesterol levels are associated with an increased risk of cancer [26-29]. Therefore, patients who were prescribed of statins might have higher LDL cholesterol level, which might be associated with decreased cancer risk.

Another limitation of our study is that due to retrospective nature of the study, the interval of endoscopy was not controlled. In Korea and Japan, endoscopy is recommended as a nationwide cancer screening program after age of 40. In this particular cohort, the median time for endoscopy interval (2 years) and mean duration between the date of entry into the diabetes cohort and gastric cancer index (673 days) are nearly the same, suggesting that the possibility of preexisting cancer at the time of diabetes cohort registry cannot be excluded. There was no difference in endoscopy intervals between statin user and non-statin user in this cohort. In addition, the drug use in this study was defined as any periods before diagnosis of gastric cancer and therefore any periods after the diagnosis was classified as non-use of statins. Our definition of use of statins presumes that gastric cancer does not occur after the use of statin. Hence, longer duration of use of statins itself was related with less chance of being diagnosed with gastric cancer. Thus, testing the duration-effect relationship may not mean more than the overall effect of statin use on the risk of gastric cancer. To further validate the impact of duration of statin use on gastric cancer incidence would be to validate the “exact case control study” using statins and their effect on cardiovascular disease or coronary artery disease [30].

Statin use was not significantly associated with other variables such as grade of differentiation, smoking status, location, or the presence of *Helicobacter pylori*. However, gastric cancer found in statin users had an increased likelihood for localized disease (P = 0.040). One of the conceivable reasons for this type of finding would be that a statin user might have been more compliant for gastroscopy screening, which led to early detection. In addition, the survival of gastric cancer patients who had used statins for more than 6 months demonstrated favorable survival (5-year OS, 89.3% vs. 73.0%; statin user vs*cpg* non-user; P=0.0873, Figure 1) when compared with non-statin users. The impact of statin use on survival of gastric cancer needs to be externally validated in order to draw a more definitive conclusion.

## Conclusion

In this study, we conducted a large exact-matched case–control study in patients with diabetes. We demonstrated that statin use may considerably reduce the risk of gastric adenocarcinoma. Although external validation is needed, a lower incidence of gastric cancer was evident in statin users. The anti-tumor effect of simvastatin as a chemopreventive agent and/or anti-tumor agent will be evaluated through ongoing trials (i.e., NCT# 00944463, NCT# 01099085).

## Competing interests

The authors declare that they have no competing interests.

## Authors’ contributions

JL, SHL drafted the manuscript. KHR, SYO, SWK participated in the design of the study and performed the statistical analysis. WK conceived of the study and participated in its design and coordination. All authors read and approved the final manuscript.

## Funding resources

This study was supported by a grant of the Korean Health Technology R&D Project, Ministry of Health & Welfare, Republic of Korea (A102166).

## Pre-publication history

The pre-publication history for this paper can be accessed here:

http://www.biomedcentral.com/1471-2407/12/596/prepub
